# Preimplantation Genetic Testing (PGT) and Prenatal Diagnosis of Schaaf-Yang Syndrome: A Report of Three Families and a Research on Genotype–Phenotype Correlations

**DOI:** 10.3390/jcm12041688

**Published:** 2023-02-20

**Authors:** Naixin Xu, Weihui Shi, Xianling Cao, Xuanyou Zhou, Hefeng Huang, Songchang Chen, Chenming Xu

**Affiliations:** 1 Obstetrics and Gynecology Hospital, Institute of Reproduction and Development, Fudan University, Shanghai 200030, China; 2 Shanghai Key Laboratory of Embryo Original Diseases, Shanghai 200030, China; 3 International Peace Maternity and Child Health Hospital, School of Medicine, Shanghai Jiao Tong University, Shanghai 200030, China; 4 Research Units of Embryo Original Diseases, Chinese Academy of Medical Sciences (No. 2019RU056), Shanghai 200030, China

**Keywords:** Schaaf-Yang syndrome, *MAGEL2*, preimplantation genetic testing, prenatal diagnosis, genotype–phenotype correlation

## Abstract

Schaaf-Yang Syndrome (SYS) is a genetic disorder caused by truncating pathogenic variants in the paternal allele of the maternally imprinted, paternally expressed gene *MAGEL2* and is characterized by genital hypoplasia, neonatal hypotonia, developmental delay, intellectual disability, autism spectrum disorder (ASD), and other features. In this study, eleven SYS patients from three families were enrolled and comprehensive clinical features were gathered regarding each family. Whole-exome sequencing (WES) was performed for the definitive molecular diagnosis of the disease. Identified variants were validated using Sanger sequencing. Three couples underwent PGT for monogenic diseases (PGT-M) and/or a prenatal diagnosis. Haplotype analysis was performed to deduce the embryo’s genotype by using the short tandem repeats (STRs) identified in each sample. The prenatal diagnosis results showed that the fetus in each case did not carry pathogenic variants, and all the babies of the three families were born at full term and were healthy. We also performed a review of SYS cases. In addition to the 11 patients in our study, a total of 127 SYS patients were included in 11 papers. We summarized all variant sites and clinical symptoms thus far, and conducted a genotype–phenotype correlation analysis. Our results also indicated that the variation in phenotypic severity may depend on the specific location of the truncating variant, suggestive of a genotype–phenotype association.

## 1. Introduction

A group of imprinted genes necessary for typical mammalian neurodevelopment can be found on chromosome 15q11-q13 [1]. Prader–Willi syndrome (PWS, OMIM #176270) is caused by paternal deletions of this area [2]. Truncating variants in *MAGEL2*, one of the paternally expressed genes in this region, were shown to induce Schaaf-Yang syndrome (SYS, OMIM #615547). The first four patients with truncating viariants in the paternal copy of *MAGEL2* were described by Schaaf et al. in 2013 [3]. These patients were referred to as having a “PW-like syndrome” because several of their phenotypic traits, such as hypogonadism, neonatal hypotonia, feeding issues, developmental delay, and intellectual disability, mirrored those of patients with PWS [3]. Their distinctive phenotypic profile can be recognized more accurately as there are more individuals with pathogenic truncating variants of *MAGEL2*. Patients with SYS had higher rates of autism spectrum disorder (ASD) than those with PWS [4,5]. Truncating variants in *MAGEL2* cause severe arthrogryposis with respiratory distress that results in neonatal mortality. Moreover, despite a variety of widespread clinical signs, including endocrine disorders such as hypopituitarism, SYS individuals exhibit a broad phenotypic spectrum [3,4,6]. *MAGEL2* variants were recently discovered in patients with Chitayat–Hall syndrome (CHS), broadening the clinical breadth of *MAGEL2*-related illnesses. However, CHS and SYS seem to have the most common clinical features and genetic causes, so many researchers regard them as the same syndrome [7,8].

*MAGEL2* is a single-exon, highly GC-rich gene that encodes a protein involved in endosomal protein recycling and is a member of the MAGE family of ubiquitin ligase regulators [9]. As a matrilineal-imprinted gene (the maternal chromosome is methylated), *MAGEL2* is only expressed by unmethylated paternal alleles, and the individual is only impacted when the variation occurs on the paternal allele [3]. Variants in the maternal-imprinted allele can cause the symptoms to skip several generations in contrast to autosomal dominant inheritance. However, for male individuals carrying a deleterious *MAGEL2* variant, 50% of their offspring will be clinically affected [4]. Even though the majority of SYS patients are able to survive the illness, fatal cases cannot be disregarded. Neurodevelopmental delay with unexplained dyspnea is clinically extremely hazardous, leading to death in the neonatal period of SYS patients [9,10]. As SYS does not comply with Mendelian inheritance laws and the underlying pathological mechanisms and genotype–phenotype correlations remain to be elucidated, clinical suspicion of SYS in the differential diagnosis in pediatric patients with hypotonia and developmental delay/intellectual disability is not high because of the physicians’ unfamiliarity [11].

Preimplantation genetic testing for monogenic disorders (PGT-M) and prenatal diagnosis are essential for families with SYS-affected members to prevent the transmission of the variant [12,13,14]. PGT-M is a component of the in vitro fertilization (IVF) process that genetically profiles oocytes or embryos before implantation [15,16,17]. It is generally accessible for any monogenic condition when the causative variant is known [18,19,20]. Couples who decide to use PGT-M for IVF have two major challenges: identifying the disease-causing variation and performing an appropriate biopsy and subsequent genetic analysis that prevents any harm to embryo viability [21].

Here, we report on eleven patients with SYS from three Chinese families, along with the variant spectrum and phenotype profile of SYS. In addition, we performed PGT and/or a prenatal diagnosis in three couples from families who suffered from SYS and successfully prevented the transmission of the truncating variants in *MAGEL2*. Moreover, we performed a review of SYS-related literature, summarized all variant sites and clinical symptoms of SYS thus far, and conducted a genotype–phenotype correlation analysis.

## 2. Materials and Methods

### 2.1. Study Subjects

Three families with SYS were recruited from the International Peace Maternity and Child Health Hospital in this study. The study was authorized by the International Peace Maternity and Child Health Hospital, and all participants provided informed consent in accordance with ethical norms.

### 2.2. DNA Extraction and Variant Detection

These methods have been described in detail in our previous research [21].

### 2.3. In Vitro Fertilization (IVF)

Controlled ovarian hyperstimulation (COH) was conducted with human chorionic gonadotropin (hCG). Gonadotropin-releasing hormone (GnRH) antagonists constitute a multiple-dose flexible regimen for the prevention of ovarian hyperstimulation syndrome (OHSS). Intracytoplasmic sperm injection (ICSI) was performed on metaphase II (MII) oocytes, and the resultant embryos were cultured to the blastocyst stage for biopsy. The biopsied blastocysts were cryopreserved for further embryo transfer cycles utilizing the vitrification procedure with individual tubes containing single blastocysts.

### 2.4. Identity Testing and Haplotype Analysis

According to the instructions, short tandem repeats (STRs) were utilized for identity testing and detecting probable maternal contamination with an identification detection kit (R1004T; GENESKY, Shanghai, China). The general sample processing kit for gene sequencing (Yikon Genomics, Shanghai, China) was used to perform whole-genome amplification on each embryo biopsy sample according to the manufacturer’s instructions. Using a 1.5 Mb custom probe encompassing 350 kb upstream to 350 kb downstream of the *MAGEL2* gene, multiple displacement amplification (MDA) products and gDNA libraries were created and collected. Haplotype analysis was performed to confirm the carrier status in the family and the embryonic inheritance based on informative STRs that cosegregate with the detected variant and are found in the same region in both parents but are homozygous in one and heterozygous in the other.

### 2.5. Prenatal Diagnosis

Pregnancy was verified by a blood hCG level of 25 U/L 14 days after transplantation and determined by ultrasound of the fetal sac with heart rhythm in the uterine cavity 30~40 days after transplantation. In the second trimester, a prenatal diagnosis was carried out via amniocentesis. Under the direction of ultrasound, 20 microliters of amniotic fluid were taken. After the genomic DNA was collected as previously mentioned, the genotypes of the fetuses were verified by Sanger sequencing.

### 2.6. Literature Review and Genotype–Clinical Phenotype Correlation Analysis

The PubMed, EMBASE, and Web of Science databases were used to conduct literature searches. The following terms were used in the search: “Schaaf-Yang syndrome”, “Prader-Willi and Schaaf-Yang syndromes”, “*MAGEL2*”, “*MAGEL2*-related disorders”, “Chitayat-Hall syndrome”, and “Chitayat-Hall and Schaaf-Yang syndromes”. Published cases of SYS caused by *MAGEL2* gene variants, as well as information on the current patient are included in our literature review. The associations between genotype and clinical phenotype were examined after collecting the clinical symptoms, laboratory test results, imaging, and gene variant status of the patients. Two patient groups were examined to determine the relationship between genotype and phenotype: patients with the c.1996dupC (p.Gln666Profs*47) variant (n = 51) and patients with any other variant (n = 67).

### 2.7. Statistical Analysis

For statistical analysis, SPSS 26.0 was used. Continuous variables are presented as mean (standard error), and categorical variables are described as n/N(percentages). Differences between groups of continuous variables were tested using a parametric two-tailed t test or Mann–Whitney U test when the normality assumption was not met. Differences between the two groups regarding categorical variables were evaluated using Fisher’s exact test or the chi-square test when the expected count was ≥5. *p* values less than 0.05 were considered statistically significant.

## 3. Results

### 3.1. History of Three Families

**Figure 1:**
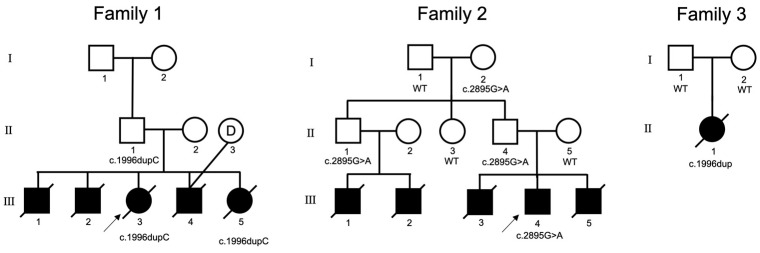
Pedigrees of three families with SYS. The arrows indicate the proband of each family. Squares represent males; circles represent females; solid symbols indicate affected patients; open symbols indicate unaffected subjects; a slash through the symbol means deceased; the circle with the letter D in the middle represents the donor egg.

Figure 1 illustrates the pedigrees of the three families with SYS included in our study. Table 1 shows the specific *MAGEL2* variant detected in the probands of these three families. The clinical characteristics of the eleven patients with SYS from the three families are summarized in Table A1.

#### 3.1.1. Family 1

The couple (II-1 and II-2) in Family 1 had five children presenting with hypotonia and abnormal mandibular development. The male children presented with gonadal dysplasia and contractures. All five children were born with hypoxia and an inability to breathe spontaneously. After rescue treatment, they showed irregular breathing, cyanosis, poor response, and a weak cry. They were admitted to the neonatal intensive care unit for respiratory and feeding support. Four children (III-1/2/4/5) died of respiratory failure shortly after birth (7–17 days). Family 1-III-3 died about 110 days after birth, and doctors suspected it might be caused by sleep apnea.

Whole-exome sequencing (WES) revealed NM019066.4(*MAGEL2*): c.1996dupC (p. Gln666Profs*47) heterozygous variant of the proband (III-3) and III-5. Variants were inherited from the unaffected father (II-1) as a heterozygous variant (c.1996dupC).

#### 3.1.2. Family 2

The proband in Family 2 (III-4) was an 11-year-old boy, diagnosed with cerebral palsy a week after birth, and complaining of feeding difficulties in the neonatal period, intellectual disability, obesity, and bulimia. A physical examination showed a micropenis. Both the older brother (III-3) and younger brother (III-5) of the proband were diagnosed with neonatal asphyxia and atelectasis after birth and died at 7–14 days of age due to respiratory failure. The parents (II-4 and II-5) were phenotypically normal. The uncle of the proband (II-1) had fathered two sons (III-1 and III-2), both of whom died of atelectasis after birth.

WES revealed the presence of NM019066(*MAGEL2*):c.2895G>A (p.W965*) heterozygous variant in the proband (III-4). The same heterozygous variant was detected in the proband’s father (II-4), uncle (II-1), and grandmother (I-2).

#### 3.1.3. Family 3

The proband (II-1) in Family 3 had pneumonia, sepsis, encephalopathy, and intracranial hemorrhage in the neonatal period. The physical examination was notable for the presence of laryngeal chondromalacia, increased muscle tone in the upper extremities, and stiffness of the middle and fourth finger joints of both hands, which could not be fully extended. Laboratory tests suggested elevated thyroid-stimulating hormone levels. Echocardiography suggested an atrial septal defect and patent foramen ovale. The proband died of apnea caused by pneumonia 32 days after birth.

Trio-whole-exome sequencing (trio-WES) revealed the presence of NM_019066.5(*MAGEL2*):c.1996dupC de novo heterozygous variants in the proband (II-1).

### 3.2. Preimplantation Genetic Testing and Prenatal Diagnosis

**Figure 2:**
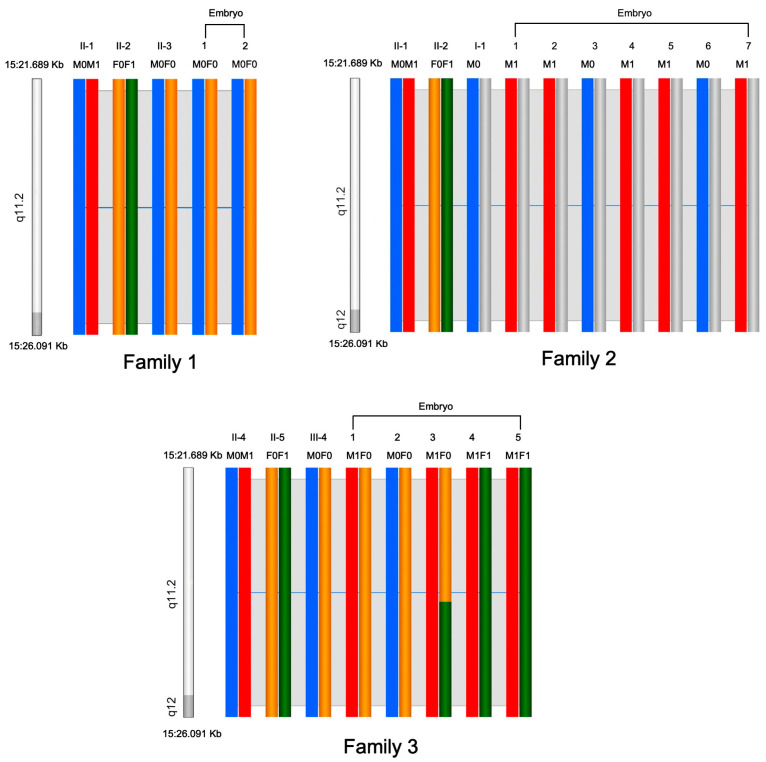
The haplotype of the MAGEL2 gene in embryos from three families. M0 means male disease-causing chromosome; M1 means male normal chromosome; F0 and F1 mean female normal chromosome.

Three couples in particular conceived via in vitro fertilization following PGT, a process whereby a 5-day-old embryo is examined in the lab to see if it possesses a specific disease-causing variation. Four PGT cycles were completed by the three couples (Family 1-II-2, Family 2-II-2, Family 2-II-5). The 14 blastocysts underwent trophectoderm biopsies and PGT-M/PGT-A (PGT for aneuploidies). The genotypes of 14 embryos were all successfully determined using haplotype analysis (Figure 2). Nine (64.2%) of the fourteen embryos were found not to harbor any *MAGEL2* variants by means of PGT-M, whereas 7 of the 14 embryos were found to be euploid by means of PGT-A (Table 2). Two embryos (Embryo 2 of Family 2-II-2, Embryo 4 of Family 2-II-5) found to be both negative for variant and euploid were used for transfer. Family 1 had only two available embryos, and both carried the disease-causing variant. The couple (Family 1-II-2) terminated PGT and embryo implantation.

**Figure A1:**
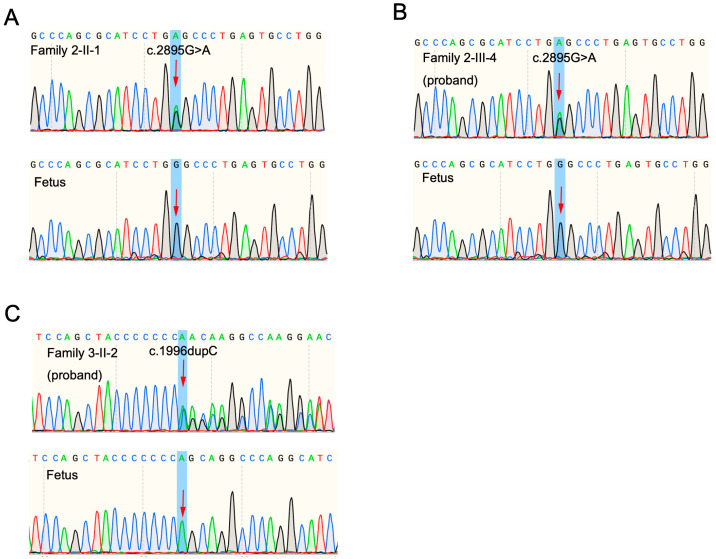
Prenatal genetic diagnosis after PGT. Sanger sequencing of the fetal samplings from amniotic fluid indicated normal genotypes in the three families. (**A**) Family 2-II-2. (**B**) Family 2-II-5. (**C**) Family 3-I-2.

The other couple (Family 3-I-2) elected to undergo a prenatal diagnosis on the fetus after the mother had conceived naturally. The results and outcomes of the prenatal diagnosis in the three families are summarized in Table 3. The prenatal diagnosis results showed that the fetus did not carry variants (Figure A1). All babies of the three families were born at full term and were healthy.

### 3.3. Literature Review

**Figure 3:**
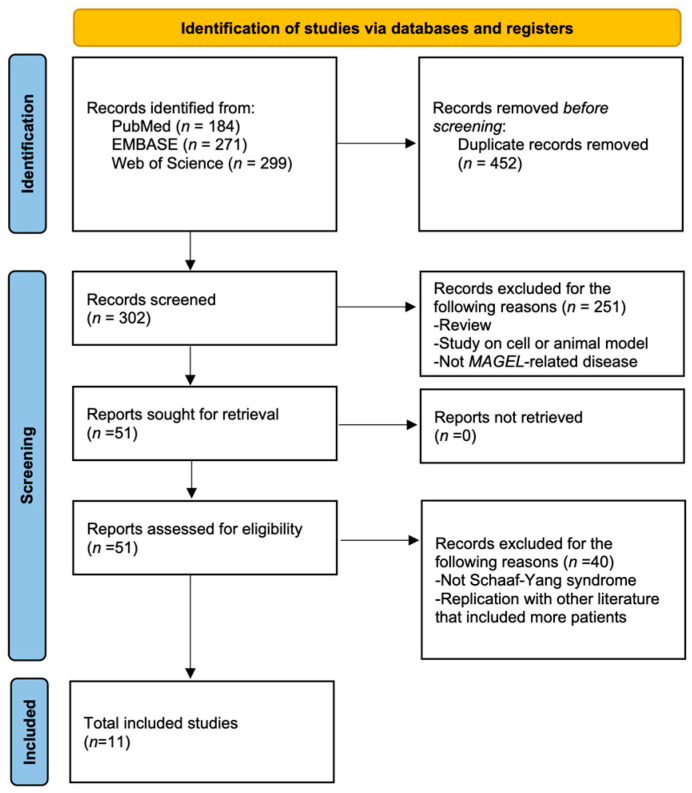
Flow diagram of the literature inclusion.

After excluding duplicate cases, 11 papers were found from the literature review in which these syndromes were discussed (Figure 3). Together with the 11 patients in our study, data from a total of 127 patients with SYS were pooled and were available for analysis. We numbered patients according to the literature sources: 1–91 [8], 92–95 [23], 96 [24], 97–102 [6], 103–104 [9], 105–108 [11], 109 [25], 110 [26], 111 [27], 112–115 [28], 116 [29] and 117–127 (New).

### 3.4. MAGEL2 Gene Variant Spectrum

**Figure 4:**
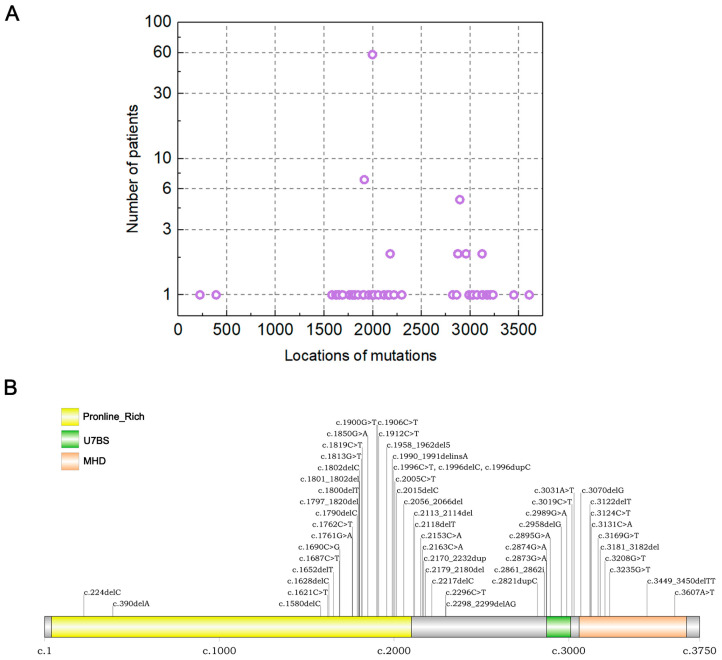
MAGEL2 gene variant spectrum in all the included literature. (**A**) Summary of the location of variants in all genes in MAGEL2. The highest peak represented the c.1996 region, which is predicted to contain a “variant hotspot”. (**B**) MAGEL2-specific variant types.

Figure 4 shows the *MAGEL2* gene variant spectrum in the included 127 SYS patients. We confirmed the most common variant as c.1996dupC. Most of the variants were present in the second half of the gene after c.1580, where most cases were found in nucleotides c.1990–c.1996, a region that represents a variant hotspot. The c.1996dupC variant represents the most prevalent variant with the largest number of individuals.

### 3.5. Clinical Phenotype Profile

Table 4 summarizes the clinical characteristics and related examinations of the included 127 SYS patients. The proportions of male and female patients were roughly the same.

It is worth noting that in terms of prenatal symptoms, a fetus with SYS rarely has characteristic imaging features. A small proportion of pregnant women experience decreased fetal movements and polyhydramnios. Patients with SYS in the perinatal period are more symptomatic, and those who die in the neonatal period comprise the highest proportion of all SYS deaths. Hypotonia (103/114, 90%) and poor suck (93/104, 89%), as well as feeding issues (98/116, 84%), are the most common symptoms in the perinatal period. In addition, approximately 70% (71/102) of patients have respiratory distress/defects, which lead to a higher rate of intubation (60/96, 63%) and mechanical ventilator use (63/104, 61%). The majority (112/128, 88%) of SYS patients have characteristic contractures of the fingers, and approximately half of the patients (28/54, 52%) have small hand features. In addition, scoliosis (38/94, 40%) and kyphosis (12/69, 17%) are also present in SYS patients.

Data are shown as n/N (%). Many patients with SYS have digestive symptoms, such as gastroesophageal reflux (40/84, 48%) and chronic constipation (49/83, 59%). On the assessment of the endocrine system, the most prominent symptom of SYS patients is short stature (29/44, 66%) due to growth hormone deficiency. Hypogonadism (44/103, 43%) is also present. In addition, hyperphagia (17/65, 26%) and excessive weight gain (23/90, 26%) are present after six years of age in a small percentage of patients.

Developmental delay (106/110, 96%) is present in the vast majority of patients with SYS. Approximately 67% (37/55) of the patients are diagnosed with autistic spectrum disorder. A small number of patients also have other psychiatric disorders, including attention deficit disorder, obsessive compulsive disorder, and some self-injurious behaviors.

Sleep apnea was present in 66 of 94 SYS patients (70%). Abnormal sleep cycles were present in a small proportion of SYS patients. Regarding the imaging findings, echocardiography abnormalities and brain MRI abnormalities were present in 18 of 31 patients. Hypopigmentation (4/32, 13%) was present in a small percentage of patients. Eye anomalies were present in 28 of 53 patients (53%).

### 3.6. MAGEL2 Genotype–Clinical Phenotype Correlation Analysis

In this study, we also contrasted the phenotypes in two groups of patients based on the sites of their *MAGEL2* variants: individuals with any variant other than c.1996dupC (n = 67) and individuals with the c.1996dupC variant (n = 51) (Table 5). Compared with patients with any variant other than c.1996dupC, respiratory distress/defects and intubation were more common symptoms in patients with the c.1996dupCvariant (81 vs. 56%, *p* value: 0.008; 73 vs. 47%, *p* value: 0.011). Among individuals with the c.1996dupC variant, 32 of 44 individuals (73%) used a mechanical ventilator compared to 22 of 47 individuals (47%) without the c.1996dupc variant (*p* value: 0.012). We also found a substantial enrichment of four symptoms in patients who harbored 1996dupC variants in comparison with patients who harbored any other variant (poor suck in infancy [*p* value: 0.009]; dysphagia [*p* value: 0.005]; use of nasogastric tube [*p* value: 0.00004]; use of G tube [*p* value: 0.005]). In the patients with the c.1996dupC variant, contractures were more common than in those with any variant other than c.1996dupC (98 vs. 82%, *p* value: 0.038). There was also a difference in the endocrine profile between the two groups, and endocrine symptoms seemed to be milder in patients with the c.1996dupC variant. Among those with a c.1996dupC variant, 5 of 40 individuals (13%) displayed excessive weight gain, while 18 of 49 individuals (37%) without a c.1996dupC variant were similarly affected (*p* value: 0.009). Those with *MAGEL2* variants other than c.1996dupC variant were more likely to have hyperphagia; there were 13 of 37 (35%) in the former case compared to 4 of 34 (12%) in the case of variants of c.1996dupC (*p* value: 0.021). In addition, there was a significant relationship between the number of symptoms and the presence of the 1996dupC variant when compared to other variants (mean number of symptoms in patients with c.1996DupC = 13.6, SE = 0.57; mean number of symptoms in patients without c.1996dupC = 11.5, SE = 0.54; *p* value: 0.012).

## 4. Discussion

The eleven patients from three families in our case series all harbored *MAGEL2* variants and had overlapping clinical presentations, including multiple congenital anomalies, contractures, respiratory stress, and micropenises in male infants. Most patients died in the neonatal period from respiratory failure or neonatal pneumonia. In childhood, surviving patients presented with developmental delay, obesity, and bulimia. Of note is that congenital heart disease (atrial septal defect and patent foramen ovale) was present in the affected child (Family 3-II-1) with the c.1996dupC variant, which has been less commonly reported in related variants and only mentioned in one case report [9]. Since the disease does not follow Mendelian inheritance laws and the patients did not undergo genetic screening and diagnosis in time, both Family 1 and Family 2 in our study had a history of recurrent birth defects and neonatal deaths. As SYS is a rare autosomal dominant and matrilineal-imprinted neurodevelopmental illness, when the mutant gene is inherited from the father, the likelihood of the disease emerging in the progeny of an affected individual is estimated to be approximately 50%. De novo variants, which account for 50% of cases, have a 2–3% likelihood of recurrence [30]. It is consequently critical in such circumstances to be able to provide PGT and a prenatal diagnosis to prevent the recurrence of SYS in the affected families. In families that underwent PGT-M, informative STRs were distributed from upstream to downstream of the *MAGEL2* gene, ensuring that any recombination would be identified. We determined each embryo successfully. Family 1-II-2 had only two available embryos, and both carried the disease-causing variant, so PGT and embryo implantation were terminated. We successfully performed PGT and/or a prenatal diagnosis in another three couples and prevented the transmission of truncating variants in *MAGEL2*.

**Figure A2:**
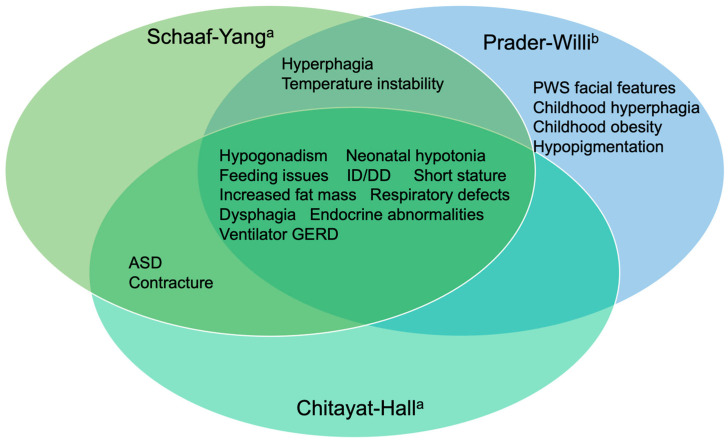
Venn diagram comparing the clinical presentations of Schaaf-Yang syndrome (SYS), Prader–Willi syndrome (PWS) and Chitayat–Hall syndrome (CHS). ^a^, disorder defined by truncating variants of the *MAGEL2* gene; ^b^, disorder defined by gene deletions in the 15q11-q13 chromosomal region.

In this paper, we have summarized the data of 127 patients from around the world diagnosed with truncating variants in the *MAGEL2* gene to increase clinicians’ understanding and the diagnostic ability of the disease. In terms of prenatal symptoms, ultrasound of the SYS fetus rarely yields characteristic ultrasound images, and only a small number of fetuses may have nonspecific features, such as polyhydramnios or decreased fetal movement. This poses a particular difficulty in the diagnosis of SYS by routine prenatal testing. In the neonatal period, the most prominent and contributing symptom to the highest case fatality rate is respiratory defects, which in turn also leads to an elevated rate of intubation and ventilator use. Respiratory disorders may persist into infancy and adulthood, as sleep apnea is often reported as a problem that must be handled throughout one’s life. Hypotonia is almost universally found (90%) in patients with SYS and contributes to many clinical manifestations of the disease, including feeding difficulties and poor suck in infancy, so a nasogastric tube or G tube is needed. The most prominent and exclusive dysmorphic feature of patients with SYS is contractures (88%), which is also a symptom that distinguishes SYS from PWS [23,31]. Short stature, increased fat mass, and low IGF-1 levels are common in SYS patients, indicating a growth hormone deficiency similar to PWS [32]. Hypogonadism is a consistent finding in both males and females with SYS [4,33]. Furthermore, patients with truncating variants in *MAGEL2* have a higher prevalence (67%) of autism spectrum disorder (ASD) than patients with classical PWS [31,34,35]. In general, SYS and PWS have a considerable overlap in symptoms, which further proves that the molecular diagnosis of SYS is useful and necessary. We also summarize the overlap and differences of SYS, PWS, and CHS, with the hope that the clinical and molecular characteristics of this group of disorders will become increasingly clear (Figure A2). In addition, understanding the pathogenesis through PGT can help patients block the transmission of pathogenic *MAGEL2* variants and give birth to healthy babies.

*MAGEL2* contains a proline-rich domain (PRD), a USP7-binding segment (U7BS), and a MAGE homology domain (MHD) [36,37,38]. We summarized the truncating variants of *MAGEL2* that have been reported and found that these variants are located in the PRD most commonly, followed by the MHD and U7BS domains. The c.1996dupC variant represents the most prevalent variant, affecting the largest number of individuals. By comparing clinical characteristics between variant groups, we demonstrated further genotype–phenotype associations found in SYS. Our findings suggest that the position of the truncating variant may affect how severe a syndromic condition is since people with the c.1996dupC variant exhibit a more severe and varied phenotypic profile than people with other truncating variants; this is consistent with previous research conclusions [8,39]. Specifically, joint contractures, respiratory distress/defects, intubation and the use of mechanical ventilators, difficulty sucking in infancy, and the use of a nasogastric tube and G tube all occur at a higher prevalence in people with this duplication. Interestingly, patients with the c.1996dupC variant appear to have milder endocrine symptoms. It is important to emphasize that this conclusion needs to be taken with caution because abnormalities of the endocrine system mostly present in childhood rather than the neonatal period, and some patients with the c.1996dupC variant may not survive into childhood because of a more severe respiratory distress phenotype, thus leading to a bias that patients with the c.1996dupC variant appear to have milder endocrine symptoms. Although the pathomechanism of these variations is unknown, it is worth noting that *MAGEL2* is a single exon gene, and variants that result in a premature stop codon are unlikely to generate nonsense-mediated mRNA decay [36,40,41]. Instead, a shortened protein product is anticipated as a result of such variations. Because each truncated protein product would originate from a different site of the variant on *MAGEL2*, it is possible to hypothesize that the pathogenic effect may vary depending on the location of the variant. Further research will be required to determine the underlying pathomechanics in the varied expressivity of SYS phenotypes.

*MAGEL2* is truncatingly mutated in SYS and inactivated in PWS, but it is unclear how the loss of MAGEL2 function contributes to the pathophysiology of these disorders. MAGEL2 proteins regulate protein ubiquitination by interacting with the MHD and variable domains of E3 ubiquitin ligases and deubiquitinases to generate MAGE-RING E3 ligase complexes that serve as multifunctional hubs for the alteration of important substrates in the cell [38,42]. MAGEL2 is widely expressed in the hypothalamus and plays a vital role in a critical biological process that recycles membrane proteins from endosomes via the retromer sorting process [36]. Variants in *MAGEL2* impair its capacity to promote the retromer-dependent recycling of proteins from endosomes back to the trans-Golgi network, to enhance leptin receptor expression on cell surfaces and to modulate the ubiquitination and stability of the circadian rhythm protein CRY1 [43,44,45]. Mice with a targeted *Magel2* deletion recapitulated key elements of SYS, further suggesting that MAGEL2 plays an important role in the etiology of these disorders. Neonatal *Magel2* null mice fail to thrive, have a modest increase in embryonic mortality, and demonstrate growth retardation in early life, which is followed by weight gain after weaning, and increased obesity with disturbed metabolic and endocrine homeostasis [46,47]. The loss of Magel2 in mice leads to the reduction in male and female fertility by extending breeding intervals and early reproductive decline and termination, which is consistent with the symptoms of hypogonadism in patients with SYS [48]. Due to a blunted circadian rhythm, these mice exhibit aberrant eating behavior and general hypoactivity, which are symptoms similar to those reported in SYS patients [49,50]. The MAGEL2 protein was demonstrated to interact with and modify the activity of the major elements of the circadian clock at the cellular level, providing more evidence that MAGEL2 plays an important role in controlling the circadian rhythm [45,51].

## 5. Conclusions

In conclusion, we identified eleven patients harboring pathogenic truncating *MAGEL2* variants from three Chinese families with SYS, so the *MAGEL2* variant spectrum was enriched. Meanwhile, a prenatal diagnosis and/or targeted NGS-based PGT-M was performed to prevent the transmission of the pathogenic variants of *MAGEL2* in their families. Furthermore, this study also summarized the clinical symptoms of patients with SYS to date and analyzed genotype–clinical phenotype correlations, which helps to inform families about the range of symptoms associated with SYS and may help further elucidate SYS pathogenesis, providing benefits for clinical diagnosis and management of the disease.

## Figures and Tables

**Table 1:** MAGEL2 variant detected in probands of three families.

Family	Transcript ID	DNA_Variant	Amino Acid Changes	Classification of Variants #	Inheritance
1	NM_019066.4	c.1996dupC	p.(Gln666Profs*47)	pathogenic	Pat
2	NM_019066.4	c.2895G>A	p.(Trp965*)	pathogenic	Pat
3	NM_019066.5	c.1996dupC	p.(Gln666Profs*47)	pathogenic	De novo

# The interpretation and classification of variants were based on ACMG guidelines [22]; * Termination of protein translation; pat, paternal; mat, maternal.

**Table 2:** Summary of PGT-M/PGT-A results from three families.

Sample No.	Embryo	PGT-M Result		PGT-A Result	Outcome
		Haplotype	Sanger Sequencing		
Family 1-II-2	Embryo 1	M0	c.1996dupC	−2, −15, −12	
	Embryo 2	M0	c.1996dupC	N	
Family 2-II-2	Embryo 1	M1/	N	46, XN	
	Embryo 2	M1/	N	46, XN	Transfer
	Embryo 3	M0/	c.2895G>A	46, XN	
	Embryo 4	M1/	N	46, XN	
	Embryo 5	M1/	N	46, XN	
	Embryo 6	M0/	c.2895G>A	46, XN	
	Embryo 7	M1/	N	46, XN	
Family 2-II-5	Embryo 1	M1/	N	47, XN, +22	
	Embryo 2	M0/	c.2895G>A	46, XN	
	Embryo 3	M1/	N	46, XN, −14q21.2qter	
	Embryo 4	M1/	N	46, XN	Transfer
	Embryo 5	M1/	N	46, XN	

M0: male disease-causing chromosome; M1, male normal chromosome.

**Table 3:** Summary of the results of prenatal diagnosis in three families.

Sample No.	Gestation, Weeks	Fetus	Result	Outcome
Family 2-II-2	16 W	Family 2-III-3	N	Born healthy
Family 2-II-5	17 + 4 W	Family 2-III-7	N	Born healthy
Family 3-I-2	15 W	Family 3-II-2	N	Born healthy

**Table 4:** Patients’ symptom summary.

		Patients and Source Publication												Totals ^d^ (n = 127)
		1–91	92–95	96	97–102	103–104	105–108	109	110	111	112–115	116	New	
Sex (M/F)		N/A	4/4	0/1	5/1	0/2	3/1	0/1	0/1	1/0	1/3	1/0	8/3	44/47
Prenatal symptoms	History of polyhydramnios	N/A	N/A	N/A	N/A	0/2	1/4	0/1	0/1	0/1	1/3	1/1	0/10	N/A
	Decreased fetal movement	8/23	2/8	0/1	N/A	N/A	0/4	1/1	0/1	0/1	0/4	0/1	0/7	11/51 (22%)
Perinatal history	Temperature instability	40/60	N/A	N/A	4/6	N/A	N/A	0/1	1/1	0/1	0/4	N/A	2/7	47/80 (59%)
	Respiratory distress/defects	49/70	N/A	1/1	0/6	2/2	3/4	1/1	1/1	0/1	2/4	1/1	11/11	71/102 (70%)
	Intubation	38/64	N/A	1/1	1/6	2/2	2/4	1/1	1/1	0/1	2/4	1/1	11/11	60/96 (63%)
	Mechanical ventilator	36/64	4/8	1/1	1/6	2/2	3/4	1/1	1/1	0/1	2/4	1/1	11/11	63/104 (61%)
	Tracheostomy	13/58	N/A	N/A	0/6	0/2	0/4	0/1	1/1	0/1	2/4	0/1	0/10	16/88 (18%)
	Hypotonia	72/78	6/7	1/1	6/6	2/2	3/4	1/1	1/1	0/1	1/1	1/1	9/11	103/114 (90%)
	Feeding issues	71/80	7/7	1/1	4/6	N/A	1/4	1/1	1/1	0/1	1/4	1/1	10/10	98/116 (84%)
	Poor suck in infancy	65/72	6/7	N/A	6/6	2/2	2/4	1/1	1/1	0/1	N/A	N/A	10/10	93/104 (89%)
	Dysphagia	49/62	N/A	N/A	1/6	2/2	2/4	1/1	1/1	0/1	N/A	N/A	6/6	62/83 (75%)
	Use of nasogastric (NG) tube	45/60	4/5	0/1	1/6	1/2	2/4	1/1	1/1	0/1	N/A	N/A	9/11	64/92 (70%)
	Use of G tube	30/58	N/A	1/1	N/A	0/2	1/4	0/1	0/1	0/1	N/A	N/A	0/11	32/79 (41%)
Dysmorphic features	Scoliosis	29/54	5/8	0/1	0/6	1/2	0/4	0/1	0/1	0/1	3/4	0/1	0/11	38/94 (40%)
	Kyphosis	12/37	N/A	0/1	0/6	0/2	0/4	0/1	0/1	0/1	0/4	0/1	0/11	12/69 (17%)
	Contractures	77/90	6/7	1/1	6/6	2/2	3/4	1/1	N/A	0/1	4/4	1/1	11/11	112/128 (88%)
	Small hands	15/24	N/A	N/A	6/6	2/2	1/4	0/1	0/1	1/1	1/4	N/A	2/11	28/54 (52%)
	Small feet	12/24	N/A	N/A	N/A	0/2	1/4	0/1	0/1	1/1	1/4	N/A	2/11	17/48 (35%)
Gastrointestinal	Reflux/GERD	36/66	N/A	N/A	N/A	1/2	1/4	0/1	0/1	0/1	2/4	N/A	0/5	40/84 (48%)
	Chronic constipation	40/60	6/8	N/A	N/A	N/A	0/4	0/1	0/1	N/A	3/4	N/A	0/5	49/83 (59%)
Endocrine assessment	Excessive weight gain	15/67	2/5	N/A	3/5	N/A	0/3	1/1	0/1	1/1	N/A	N/A	1/7	23/90 (26%)
	Hyperphagia	14/56	N/A	N/A	N/A	N/A	N/A	1/1	0/1	1/1	N/A	N/A	1/6	17/65 (26%)
	Hypogonadism	29/74	3/7	N/A	4/6	N/A	1/2	N/A	1/1	1/1	1/4	1/1	3/7	44/103 (43%)
	Hypopituitarism	3/9	N/A	1/1	3/3	N/A	N/A	N/A	1/1	1/1	3/4	N/A	N/A	N/A
	Growth hormone deficiency	3/8	2/8	1/1	3/3	N/A	N/A	N/A	1/1	1/1	4/4	N/A	N/A	N/A
	Short stature	13/27	N/A	1/1	5/5	N/A	3/4	N/A	1/1	1/1	4/4	N/A	1/1	29/44 (66%)
	Increased fatty tissue	4/5	N/A	0/1	N/A	0/2	0/4	1/1	0/1	1/1	0/4	N/A	1/7	N/A
	Cortical blindness	1/2	N/A	N/A	N/A	N/A	N/A	N/A	N/A	N/A	N/A	N/A	N/A	N/A
Neurodevelopment	Developmental delay	79/83	8/8	1/1	6/6	N/A	4/4	1/1	1/1	1/1	4/4	N/A	1/1	106/110 (96%)
	Attention deficit disorder	N/A	2/5	N/A	N/A	N/A	N/A	N/A	N/A	N/A	N/A	N/A	N/A	N/A
	Obsessive compulsive disorder	N/A	2/5	N/A	N/A	N/A	N/A	N/A	N/A	N/A	N/A	N/A	N/A	N/A
	ASD diagnosis	29/39	4/7	N/A	1/1	N/A	2/4	N/A	N/A	N/A	1/4	N/A	N/A	37/55 (67%)
	Seizures	25/73	N/A	1/1	2/6	1/2	1/4	0/1	0/1	0/1	0/4	0/1	0/5	30/99 (30%)
	Self-injurious behavior	9/16	4/8	N/A	4/6 ^a^	N/A	N/A	N/A	N/A	0/1	1/4	N/A	0/1	18/36 (50%)
	Intellectual disability	N/A	5/5	N/A	5/6	N/A	4/4	1/1	N/A	1/1	N/A	N/A	1/1	N/A
Sleep problems	Abnormal sleep cycle ^b^	N/A	8/8	N/A	5/6 ^c^	N/A	N/A	N/A	0/1	0/1	N/A	N/A	0/1	N/A
	Sleep apnea	43/58	5/7	1/1	1/5	N/A	2/4	1/1	1/1	0/1	2/4	1/1	9/11	66/94 (70%)
Assistant examination	Echocardiography Findings	N/A	N/A	N/A	N/A	2/2	N/A	N/A	0/1	0/1	1/4	1/1	2/2	N/A
	Brain MRI findings	6/11	N/A	0/1	4/6	0/1	2/4	N/A	1/1	N/A	2/4	N/A	3/3	18/31 (58%)
Others	Hypopigmentation	N/A	N/A	0/1	4/6	0/2	0/4	0/1	0/1	0/1	0/4	0/1	0/11	4/32 (13%)
	Hirsutism	1/6	N/A	N/A	N/A	N/A	0/4	0/1	N/A	0/1	N/A	N/A	0/2	N/A
	Eye abnormalities	18/25	N/A	0/1	5/6	1/2	0/4	0/1	0/1	0/1	4/4	0/1	0/7	28/53 (53%)

^a^ Skin picking. ^b^ A sleeping routine that is different from the normal sleep cycle (e.g., sleeping from 2 pm to 1 am). A “reverse” sleep cycle would be the most extreme form. ^c^ Infantile lethargy, weak cry. ^d^ To avoid ascertainment bias, symptoms that have been assessed or reported in less than 25% of all patients (n = 127) are not applicable.

**Table 5:** Phenotype comparison between patients with any variant other than c.1996dupC and patients with the c.1996dupC variant.

		Patients with Any Variant Other Than c.1996dupC (n = 67)	Patients with the c.1996dupC Variant (n = 51)	*p*-Value
Prenatal symptoms	History of polyhydramnios	2/11 (18%)	1/12 (8%)	0.590
	Decreased fetal movement	3/16 (19%)	0/12 (0%)	0.112
Perinatal history	Temperature instability	23/46 (50%)	24/43 (56%)	0.583
	Respiratory distress/defects	29/52 (56%)	35/43 (81%)	0.008 *
	Intubation	26/55 (47%)	32/44 (73%)	0.0111 *
	Mechanical ventilator	22/47 (47%)	32/44 (73%)	0.012 *
	Tracheostomy	6/45 (13%)	9/52 (17%)	0.589
	Hypotonia	57/63 (90%)	45/47 (96%)	0.426
	Feeding issues	55/65 (85%)	45/50 (90%)	0.395
	Poor suck in infancy	49/57 (86%)	45/45 (100%)	0.009 *
	Dysphagia	28/44 (64%)	36/40 (90%)	0.005 *
	Use of nasogastric (NG) tube	24/51 (47%)	39/45 (87%)	0.00004 *
	Use of G tube	11/46 (24%)	21/39 (54%)	0.005 *
Dysmorphic features	Scoliosis	17/51 (33%)	19/38 (50%)	0.113
	Kyphosis	5/38 (13%)	7/35 (20%)	0.431
	Contractures	54/66 (82%)	51/52 (98%)	0.038 *
	Small hands	7/17 (41%)	7/14 (50%)	0.623
	Small feet	2/12 (17%)	4/13 (31%)	0.645
Gastrointestinal	Reflux/GERD	15/40 (38%)	25/43 (58%)	0.351
	Chronic constipation	23/44 (52%)	27/42 (64%)	0.259
Endocrine assessment	Excessive weight gain	18/49 (37%)	5/40 (13%)	0.009 *
	Hyperphagia	13/37 (35%)	4/34 (12%)	0.021 *
	Hypogonadism	25/53 (47%)	18/44 (41%)	0.537
	Hypopituitarism	7/7 (100%)	3/4 (75%)	0.364
	Growth hormone deficiency	22/43 (51%)	17/30 (57%)	0.643
	Short stature	30/48 (63%)	24/37 (65%)	0.822
	Increased fatty tissue	3/9 (33%)	0/13 (0%)	0.025 *
	Cortical blindness	0/1 (0%)	N/A (/)	/
Neurodevelopment	Developmental delay	45/53 (85%)	33/34 (97%)	0.069
	Attention deficit disorder	2/6 (33%)	N/A (/)	/
	Obsessive compulsive disorder	2/6 (33%)	1/1 (100%)	0.429
	ASD diagnosis Seizures	24/34 (71%)	14/21 (67%)	0.760
	Seizures	17/53 (32%)	12/43 (28%)	0.658
	Self-injurious behavior	7/16 (44%)	2/5 (40%)	1.000
	Intellectual disability	15/15 (100%)	3/3 (100%)	1.000
Sleep problems	Abnormal sleep cycle	11/15 (73%)	2/2 (100%)	1.000
	Sleep apnea	36/52 (69%)	27/40 (68%)	0.859
Assistant examination	Echocardiography Findings	2/4 (50%)	4/7 (57%)	1.000
	Brain MRI findings	6/11 (55%)	6/9 (67%)	0.670
Others	Hypopigmentation	4/19 (21%)	0/14 (0%)	0.119
	Hirsutism	0/6 (0%)	0/3 (0%)	1.000
	Eye abnormalities	5/14 (36%)	5/14 (36%)	1.000
Total number of symptoms		11.5 (0.54)	13.6 (0.57)	0.012 *

Statistically significant *p* values were marked in bold. Data are shown as n/N (%) or mean (SE); * *p* < 0.05.

## Data Availability

The data presented in this study are available upon request from the corresponding author.

## Appendix A

**Table A1:** Clinical characteristics of the eleven SYS patients in three families.

Patient	Sex	Age	Variant Location	Respiratory Distress	Hypotonia	Hypogonadism	Other Clinical Phenotypes
Family 1-III-1	M	7 d	c.1996dupC	Neonatal anoxia	+	+	Camptodactyly
Family 1-III-2	M	15 d	c.1996dupC	Neonatal anoxia	+	+	Camptodactyly, patent foramen ovale
Family 1-III-3	F	110 d	c.1996dupC	Recurrent apnea, respiratory failure	+	−	Camptodactyly, simian line
Family 1-III-4	M	17 d	c.1996dupC	Neonatal anoxia	+	+	Camptodactyly, simian line, small mandible, high palate arch, small tongue, low ear position
Family 1-III-5	F	9 d	c.1996dupC	Neonatal anoxia	−	−	Camptodactyly, high palate arch, low ear position, bilateral polydactyly
Family 2-III-4	M	11 y	c.2895G>A	Neonatal anoxia	−	+	Feeding difficulties, intellectual disability, obesity, bulimia
Family 2-III-1/2/3/5	M	1–14 d	c.2895G>A	Neonatal anoxia, atelectasis	+	+	-
Family 3-III-4	F	32 d	c.1996dupC	Neonatal pneumonia	−	−	Neonatal sepsis, neonatal encephalopathy, intracranial hemorrhage, camptodactyly, thyroid-stimulating hormone, atrial septal defect, and patent foramen ovale

M, male; F, female; d, days; y, years.
